# Functional and physiochemical properties of the yoghurt modified by heat lactosylation and microbial transglutaminase cross‐linking of milk proteins

**DOI:** 10.1002/fsn3.3108

**Published:** 2022-10-21

**Authors:** Narin Muhammadamin Nanakali, Jasim Muhammad Al‐saadi, Chnar Sulaiman Hadi

**Affiliations:** ^1^ Department of Food Technology, College of Agricultural Engineering Sciences Salahaddin University‐Erbil Erbil Iraq; ^2^ Department of Food Technology, College of Food Science Al‐Qasim Green University Bagdad Iraq

**Keywords:** functional properties, lactosylating, microbial transglutaminase, Millard reaction, protein cross‐linking, yoghurt

## Abstract

This study aimed to recognize the effect of Maillard reaction (MR) on the functional properties of milk proteins and the physiochemical, textural, and sensory properties of yoghurt. Heating at 100°C for 2 h increased the carbohydrate ratio in caseins, whey proteins, and total milk proteins from 2.83%, 1.93%, and 1.8% to 4.15%, 3.58%, and 5.32%, respectively. Solubility of the lactosylated caseins, whey proteins, and total milk proteins is increased at low pH values compared to that of the control caseins, whey proteins, and total milk proteins. Lactosylation at 70 and 100°C increased the emulsion activity index (EAI) of caseins at all pH values, especially at pH below 6, and this increment was higher for casein samples treated at 100°C. Foam volume of whey proteins and total milk proteins also increased for samples lactosylated at 100°C compared to control samples. The combination of heating and microbial transglutaminase (MTGase) had a synergistic and enhancing effect on the pH values of yoghurt samples, especially in yoghurt samples produced by whole milk protein compared to control samples. Viscosity and hardness of yoghurt samples were enhanced by heat lactosylation, MTGase treatment, and also storage for 21 days at 7 ± 1°C.

## Graphical abstract

Heat treatment of milk proteins in the presence of lactose increased the lactose ratio in these proteins and increased the cross‐linking between these proteins, at 100°C heat treatment of all milk protein samples was more effective than 70°C for 2 h. Lactosylation of caseins, whey proteins, and total milk proteins through Maillard reaction (MR) increased their solubility at low pH values. Lactosylation of caseins and total milk proteins by a prolonged heat treatment increased their emulsion activity index (EAI), the viscosity, hardness, water‐holding capacity (WHC), and sensory properties of yoghurt during storage for 21 days.

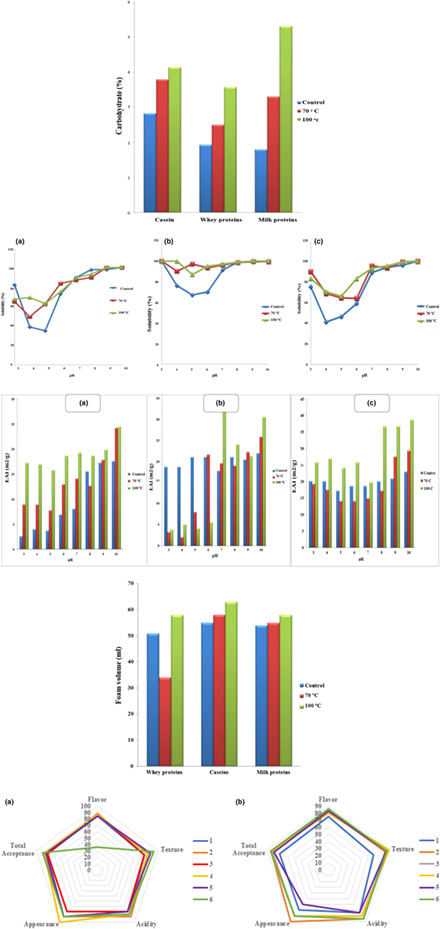

## 1 INTRODUCTION

The heat treatment of milk at high temperatures (above 90°C) induces several changes including: dephosphorylation and proteolysis of individual caseins, increase in average micellar diameter, disaggregation or aggregation of micelles, dissociation of κ‐casein from the micelle, alterations in the calcium distribution between colloidal and soluble phases and Maillard reaction (MR; Al‐Saadi et al., 2013). The term “Maillard reaction” or the so‐called nonenzymatic browning was first described by the French chemist Louis Maillard as a condensation reaction between amines and carbonyl compounds, especially reducing sugars. The MR occurs in heated and stored foods containing protein and carbohydrate such as dairy products (Fay & Brevard, 2005). Many factors, such as pH, humidity, temperature, and the nature of the reactant, are effective on the MR. Meanwhile, humidity and temperature are the most important factors during food processing and storage (Alfayadh et al., 2018).

Microbial transglutaminase (MTGase) enzyme is generally known as a protein modifier. Transglutaminase (TGase) enzyme has the ability of cross‐linking the amino acid residues of protein‐bound glutamine as an acyl donor and lysine as an acyl receptor to form both intra‐ and intermolecular isopeptide bonds in different food proteins. The application of MTGase in dairy‐based food products to improve functional and processing characteristics is increasing (Romeih & Walker, 2017). The basic mechanism of this process involves polymerization, which changes the hydrophobicity of molecules. This enzyme increases protein solubility and thereby improves other functional properties such as gelation, emulsification, viscosity, foaming, and water‐holding capacity (WHC). Despite the large number of studies on the benefits of enzymes to modify the protein function in a variety of food systems, the mechanisms are less well studied (Gaspar & de Góes‐Favoni, 2015).

Protein cross‐linking is defined as a reaction between amino acids in different polypeptide chains or a polypeptide chain to form covalent cross‐links. This reaction is likely to occur under severe food processing conditions such as alkaline pH, presence of oxygen, high temperature, and uncontrolled enzyme activity. Modification of protein structure and functional and nutritional properties of products are among the results of this reaction.

Yoghurt is a popular fermented dairy product widely consumed around the world, which is prepared from the lactic acid fermentation of milk with a starter culture containing *Streptococcus salivarius* subsp. *thermophilus* and *Lactobacillus delbrueckii* subsp. *bulgaricus*. It can be stated that these two bacteria play a role in yoghurt production by acidifying milk and synthesizing aromatic compounds (Sahan et al., 2008; Serra et al., 2009).

Hiller and Lorenzen (2011) made yoghurt from milk proteins modified with MR and they found that there was 58% decreased whey drainage in comparison with yoghurt made from untreated milk protein. In terms of sensory characteristics, milk proteins modified with MR have an effect on lactic acid fermentation, yoghurt gel formation, and microbial production of aromatic active compounds, thereby improving the texture, mouthfeel, and aroma of the product. De Goes‐Favoni and Bueno (2014) reported that MTGase reacts with both commercial α‐lactalbumin and β‐lactoglobulin without requiring any pretreatment, but the progress of the cross‐linking reaction is dependent on a high concentration of this enzyme and a long incubation time (50 MTG units/g protein for 4 h).

Also, Cozzolino et al. (2003) have claimed that the accessibility of TGase to different types of caseins individually depends on their location in the micelle. For example, on the surface of the micelles, κ‐caseins and then β‐caseins are dominant, but in the center of the micelles, αs‐caseins are more concentrated. Guyot and Kulozik (2011) used TGase‐treated skimmed milk powder (TG‐SMP) as protein enrichment for yoghurt production, where they prepared TG‐SMP by the spray‐drying process with preheated condensed skimmed milk with 8% protein content in which natural TGase inhibitors were inactivated. MTGase was added and then heat treated before the spray‐drying process to inactivate the enzyme. It was found that the viscosity of produced yoghurt was almost double that of the control, which remarkably increased the WHC and gel strength. In this research, it has been tried to investigate the effect of MTGase enzyme at different temperatures on milk proteins and as a result, the physiochemical, functional, and sensory properties of yoghurt.

## 2 MATERIALS AND METHODS

### 2.1 Material

Milk was collected from 15 cows from a dairy farm unit (Department of Animal Resources, College of Agriculture, Salahaddin University). Milk powder (Al Mudhish, 26% protein, 28% fat, and 37% lactose) was purchased from the local market, which was produced by the Oman Foodstuff Factory. Microbial transglutaminase (MTGase) Activa MP was supplied by Ajinomoto Foods Europe S.A.S. ‐153, rue de courcelles, Paris, France. Lyophilized mixed starter culture containing the bacteria (*Lactobacillus delbrueckii* subsp. *bulgaricus* and *Streptococcus salivarius* subsp. *thermophilus*) was purchased from France Rhodia Food Co.

### 2.2 Methods

#### 2.2.1 Preparation of milk protein

##### Isolation of milk proteins

Separation of milk proteins was done by mixing skimmed milk with trichloroacetic acid (TCA) (24% w/v) 1:1 for 30 min. Milk protein precipitate was collected by filtration through Whatman No. 1 filter paper and the supernatant layer was separated. Milk protein precipitate was washed twice with 12% TCA, adjusted to pH 7 by NaOH (2 M), and dissolved in distilled water. Then, it was dialyzed against water for 48 h and dried by a freeze dryer (Al‐Saadi et al., 2013).

##### Preparation of sodium caseinate

Skim milk was acidified to pH 4.6 with hydrochloric acid (HCl) (1 M) while stirring at 20°C. The mixture was filtered using Whatman No. 1 paper after settling for 20 min. Distilled water was used to wash the precipitated casein and it was dissolved by adding NaOH (1 M) until the pH reached 6.8 and precipitated again. Then, the caseinate was redissolved and dried using a freeze dryer (Al‐Saadi & Deeth, 2011).

##### Preparation of whey proteins

After isoelectric precipitation of casein, whey was collected and mixed with TCA (24%) 1:1 for 30 min. The whey precipitate was collected by filter paper and washed twice with 12% TCA, and dissolved in distilled water by adding NaOH (2 M) until pH 7 was reached. Then, it was dialyzed with tap water for 48 h and finally dried with a freeze dryer (Al‐Saadi et al., 2013).

##### Modification of proteins by MR

Freeze‐dried milk protein, sodium caseinate, and whey proteins were mixed with lactose (w/w = 1/2) and heated in oven at 70°C or 100°C for 2 h and 68% relative humidity (RH) to induce MR (Hiller & Lorenzen, 2010). After heat treatments, proteins were dissolved in water and the solutions were dialyzed against tap water for 24 h to remove the excess of lactose and then freeze‐dried. Milk powder (26% protein, 28% fat, and 37% lactose) was treated under the same conditions (time, temperatures, and humidity). Milk powders were stored in the freezer until used in yoghurt production (de Oliveira et al., 2016).

##### Total protein determinatian

The nitrogen content in milk sample was estimated by Kjeldahl's method (AOAC, 2000). The correction factor of nitrogen was 6.38.

##### Lactose determination

Phenol–sulfuric acid method was used to estimate carbohydrates’ concentration in proteins as follows: 1 ml of protein sample was mixed with 1 ml of phenol solution (5%) in a test tube and after mixing with 5 ml of sulfuric acid (18 M) and constant agitation, it was stored for 10 min. After shaking, the tube was placed in a water bath at 25°C for 20 min and the optical absorption of the mixture was measured at 490 nm. Carbohydrate concentration was calculated from the standard curve of lactose (20–80 μg/ml) under the same conditions (Zhong et al., 2006).

#### 2.2.2 Sodium dodecyl sulfate‐polyacrylamide gel electrophoresis

An appropriate amount of separating gel was prepared in a small beaker, then the specific volume of ammonium persulfate (AP) and tetramethyl ethylenediamine (TEMED) was added. The gel solution was pipetted into the gap between the glass plates of gel casting. The blank space was filled with ethanol and allowed 30 min for a complete gelation. Ten percent AP and 1% TEMED were added to the prepared appropriate amount of stacking gel. The ethanol was poured out and the separating gel solution was pipetted into the gap in the first step and the comb was inserted and allowed 30 min to undergo polymerization. The sample was mixed with the sample buffer (1:1) and the obtained mixture was boiled for 5 min in a water bath and then 10 μl of this mixture was poured into the gels' hole. The gels were run under a constant voltage of 200 V for about 3.5 h. Electrophoresis was carried out on a vertical slab unit (BDH gel tank) and Consort power supply. The gel was stained using Coomassie Brilliant Blue G‐250 solution (0.25 g stain in 8:46:46 acetic acid:methanol:water) for 24 h and destained using 5% acetic acid solution (Al‐Saadi & Deeth, 2011).

#### 2.2.3 Functional properties of milk protein

##### Solubility

Stock solutions (0.1% in 0.15 M NaCl, pH 7) of the total milk protein, casein, and whey protein were adjusted to the appropriate pH (10–3) with 0.1 N HCl or 0.1 N NaOH and centrifuged at 12,000 *g* to 15 min at 25°C. The protein concentration of the supernatant was measured by recording the absorbance value at a wavelength of 280 nm. Finally, the solubility was determined as the percentage of protein in the solution (Al‐Saadi & Deeth, 2011).

##### Foaming

The gas‐sparging method by Waniska and Kinsella (1979) was used to measure the foaming properties of milk protein, casein, and whey protein. Fifteen milliliters of samples (0.l% in 0.15 M NaCl, pH 7) was poured in a column (1.6 × 70 cm) and nitrogen gas was sprayed from the bottom of the column for 2 min with a flow rate of 30 ml/min. The foam height was determined instantly after stopping the gas flow.

##### Emulsion activity index measurement

The method of Pearce and Kinsella (1978) was used to determine the emulsion activity index (EAI). Triple emulsions of each sample (0.1% in 0.15 M NaCl, pH adjusted from 3 to 10 with 0.1 N HCl or 0.1 N NaOH) were prepared using 10 ml of sample and 0.6 ml of corn oil. An emulsion was prepared by mixing these ingredients for 1 min at room temperature. A 0.1% sodium dodecyl sulfate (SDS) solution was used to dilute 0.2 ml of the emulsion (1–250). A spectrophotometer at a wavelength of 500 nm was used to measure the turbidity of the emulsion. Emulsion activity index, which is defined as the immobilized interfacial area per unit protein weight (m^2^/g), was calculated using Equation (1):

(1) $$\mathrm{EAI}\;\left(m^{2}/g\right)=2\times 2.303\times A_{0}\times \text{dilution}/\left[C\times \left(1-ɸ\right)\times 10^{4}\right]$$

where *C* is the concentration of protein before emulsification (g/ml), *F* is the oil volume fraction (v/v) of the emulsion (ϕ is equal to 0.25 here), dilution is equal to 100, and *A*_0_ shows the absorbance at time (*t* = 0) at 500 nm.

#### 2.2.4 Yoghurt preparation and physiochemical analysis

##### Yoghurt manufacture

Whole milk powder samples (control, whole milk powder lactosylated at 70°C, and whole milk powder lactosylated at 100°C) were dissolved in water (13%) and heated to 90 ± 2°C for 5 min and then cooled to 42°C. Milk samples were divided in two parts and MTGase was added (8 U/G protein) to one part of them. Preactivated yoghurt starter (3%) was added to milk samples and the inoculated milk sample was distributed in 100 ml plastic cups and incubated at 42 ± 2°C for 3–4 h until the pH decreased to 4.6 and stored at 7 ± 1°C for 21 days (Belitz et al., 2009).

##### pH determination

An electronic digital pH meter (InoLab WTW Series 720, Germany) was used to determine the pH of milk and yoghurt. Also, calibration of the pH meter was carried out by a buffer solution of pH 4 and 7 (Bahrami et al., 2017).

##### Viscosity determination

Rawson and Marshali (2007) method with some modification was used to measure viscosity. The gel was broken by stirring with a glass rod (10 times clockwise; 10 times anticlockwise). Also, a Brookfield viscometer (model DV‐E; Brookfield Engineering Laboratories) with spindle No. 7 was used to measure the rotational viscosity measurements. All measurements were carried out at room temperature with 100 rpm (revolutions per minute) for 1 min (Hassanzadeh et al., 2016).

##### Spontaneous whey separation determination

A procedure described by Amatayakul et al. (2006) was used to determine the spontaneous whey separation (SWS). A cup of the set yoghurt was picked up from the refrigerator at 7 ± 1°C and a needle connected to syringe was used to withdraw the liquid whey from the surface of the sample and the cup of yoghurt was weighed again. This process took less than 10 s to prevent further leakage of whey from the curd.

##### WHC determination

The WHC of yoghurt was determined, as described by Harte et al. (2003). In this method, 10 g of yoghurt was centrifuged at 5000 *g* for 10 min at a temperature of 7 ± 1°C. The obtained supernatant was carefully weighed to determine the amount of separated water (Equation 2).

(2) $$\mathrm{WHC}\%=\left[1–\left(w_{2}/w_{1}\right)\right]\times 100$$

where *w*_1_ is the weight of yoghurt used and *w*_2_ is the weight of whey after centrifugation (Shori et al., 2013).

##### Texture determination

The evaluation of textural properties was conducted using a texture analyzer (CT3[4500], Brookfield Engineering Laboratories). For this purpose, an artificial plastic cylinder (20 mm in diameter) was inserted into each product to a depth of 20 mm with 5.0 g trigger and speed of 1 mm/s (Jasim & Al‐Saadi, 2020).

##### Sensory evaluation

Sensory evaluation was carried out for yoghurt produced by cow milk powder to evaluate the total acceptance of yoghurt sample by 12 panelists from the staff of Food Technology Department, Agriculture Technical College of Halabja. Sensory evaluation form was used for yoghurt samples on the first day and 21 days stored at 7 ± 1°C. Finally, sensory properties, including flavor, texture, acidity, appearance, and total acceptance yoghurt samples, were evaluated based on 100 points (Alfayadh et al., 2018).

### 2.3 Statistical analysis

One‐way analysis of variance (ANOVA) was used to determine the significance of the studied factors, and Tukey' s test was used to compare the mean of the treatments (*α* = .05) with MiniTab software (version 17). Also, Microsoft Office‐Excel was used to draw the graphs.

## 3 RESULTS AND DISCUSSION

### 3.1 Effect of heat treatments on lactose conjugation to milk proteins

**FIGURE 1:**
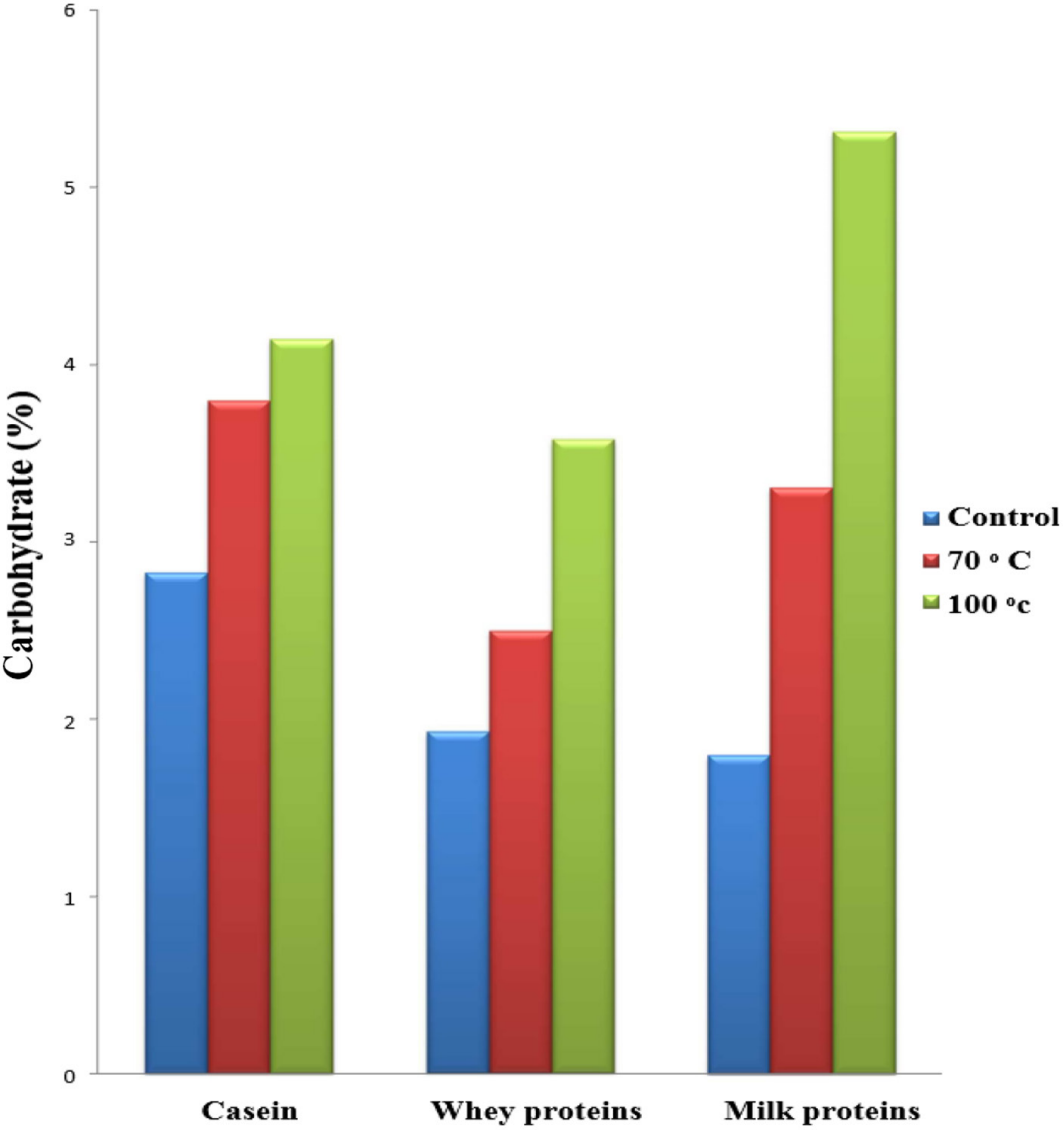
Effect of heating temperatures (70 and 100°C for 2 h) on carbohydrate concentrations in caseins, whey proteins, and total milk proteins.

Heat treatments (70 and 100°C) for 2 h were used to study their effect on the conjugation of lactose to caseins, whey proteins, and total milk proteins. Figure 1 shows the effect of heat treatments on lactose to milk proteins ratio. Lactose to milk proteins ratios were 2.83%, 1.93%, and 1.8% in control caseins, whey proteins, and total milk proteins, which increased to 3.8%, 2.5%, and 3.31% after heating at 70°C for 2 h, respectively, and heating at 100°C for 2 h increased the lactose ratio in caseins, whey proteins, and total milk proteins to 4.15%, 3.58%, and 5.32%, respectively. From the above results, it can be noted that the lactosylation of caseins, whey proteins, and total milk proteins increased with the increase of heat temperatures and this may be due to the increase of MR intensity with raising the heat temperatures (Al‐Saadi & Deeth, 2011). Besides that, Figure 1 shows that the lactosylation ratio of caseins and total milk proteins was higher than that of whey proteins and this may be related to the random and open conformation of caseins which make them more capable of reacting with lactose in comparison to the whey proteins which seem to be compacted with a globular conformation.

The sodium dodecyl sulfate‐polyacrylamide gel electrophoresis (SDS‐PAGE) analysis of the milk protein samples treated with different heat treatments is shown in Figure S1 for casein, whey protein, and total protein. The electrophoretic configurations indicate the formation of new bands resulting from cross‐linked proteins involving dehydroalanine in the treatment of whey proteins. Maillard reaction (MR) products are responsible for the formation of polymeric milk proteins visible in SDS‐PAGE. With raising temperature, the percentage of cross‐linked proteins is increased because this improves the formation of dehydroalanine. In Figure S1 it can be observed that the formation of high‐molecular‐weight dehydroalanine aggregates these proteins during heat treatments which are formed by the cross‐linking of milk proteins as a result of MR (Pesic et al., 2011) and this cross‐linking of proteins was increased with enhancing the heating temperatures.

### 3.2 Functional properties of lactosylated milk proteins

#### 3.2.1 Solubility of lactosylated caseins

**FIGURE 2:**
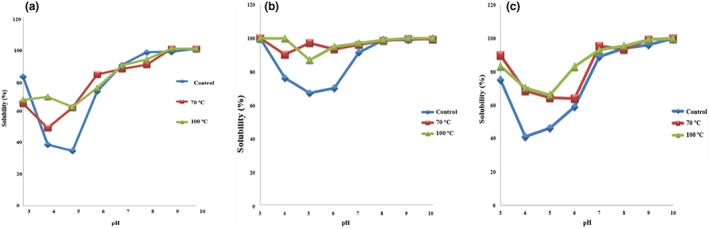
Effect of pH on the solubility of control (♦), lactosylated at 70°C for 2 h (■), and lactosylated at 100°C for 2 h (▲) in 0.15 M NaCl for casein (a), whey protein (b), and total milk protein (c).

The solubility characteristic of proteins can be introduced as an index to optimize the effects of pH on them and also to indicate the potential advantages and disadvantages of using them in food. Figure 2 shows the solubility of control caseins, caseins heat treated at 70°C for 2 h, and caseins heat treated at 100°C for 2 h in pH values between 3 and 10. The solubility of control caseins was low at pH values close to their isoelectric point (4.6) and their solubility increased gradually with the increase of pH until they reached 100% at pH values of 8 and more. This result is in accordance with the recorded results by Al‐Saadi and Deeth (2011) who found that the solubility of sheep milk caseins was low at pH values close to their isoelectric point.

Modification of casein by TGase in the absence of glucosamine only caused the formation of cross‐linking of protein molecules and the formation of some large peptide polymers. Also, the high molecular masses and extended protein structures of large polymers improve some of the functional properties of cross‐linked casein. It has been found that TGase treatment, especially at high acidic pH (pH 2) and pH above 7.0, caused a gradual and significant decrease in the solubility of proteins isolated from beans (Jiang & Zhao, 2011). Lactosylation of caseins through MR (70°C for 2 h and 100°C for 2 h) increased their solubility at low pH values, as shown in Figure 2. This may be related to the conjugation of lactose to casein, which increases the hydrophilic sides on caseins to make them capable to binding to water more efficiently (Hiller & Lorenzen, 2009).

#### 3.2.2 Solubility of lactosylated whey proteins

Whey proteins are a mixture of proteins that differ in their chemical and biological properties. Figure 2 shows the solubility of control whey proteins, whey proteins lactosylated at 70°C for 2 h, and whey proteins lactosylated at 100°C for 2 h. The solubility of control whey proteins was low at pH values between 4 and 6 and this may be related to the fact that the isoelectric points of the two major whey proteins (β‐lactoglobulin and α‐lactalbumin) are 5.6 and 4.3, respectively (Al‐Saadi et al., 2002). Lactosylation of whey proteins through MR led to the increase of whey protein's solubility at low pH values for both samples (heat treated at 70 and 100°C for 2 h). This may be attributed to the conjugation of carbohydrate to whey proteins through MR which increases their solubility (Pelegrine & Gomes, 2008).

#### 3.2.3 Solubility of lactosylated total milk proteins

The effect of pH on the solubility of total milk proteins is summarized in Figure 2. The solubility of control total milk proteins at pH 7 and higher was high. At acidic pH values (4–5), the solubility was low because this range of pH is close to the isoelectric point of casein, which is the major part of cow milk proteins. Heat treatment with lactose at 70 and 100°C for 2 h increased the solubility of total milk proteins at low pH values because the binding of lactose to total milk proteins increased the hydrophilic site in such proteins (Augustin & Udabage, 2007).

In accordance with these results, Jiang and Zhao (2011) compared the solubility of original casein, cross‐linked casein, and the modified product in the pH range of 2–12. Their results indicated that the three protein samples tested had an isoelectric point close to pH 4.5. Cross‐linked casein had lower solubility than original casein, which could be due to the TGase treatment. In general, the modified product was more soluble than the original casein at all pH levels tested, especially at neutral pH (6–7) and alkaline pH (8–12). The solubility of the modified product at pH 9 and pH 6 was significantly higher than that of original casein by 10.2% and 23.9%, respectively. This illustrates that glucosamine conjugation of casein can enhance the solubility of the modified product.

### 3.3 Emulsion activity index

#### 3.3.1 Emulsifying properties of casein

**FIGURE 3:**
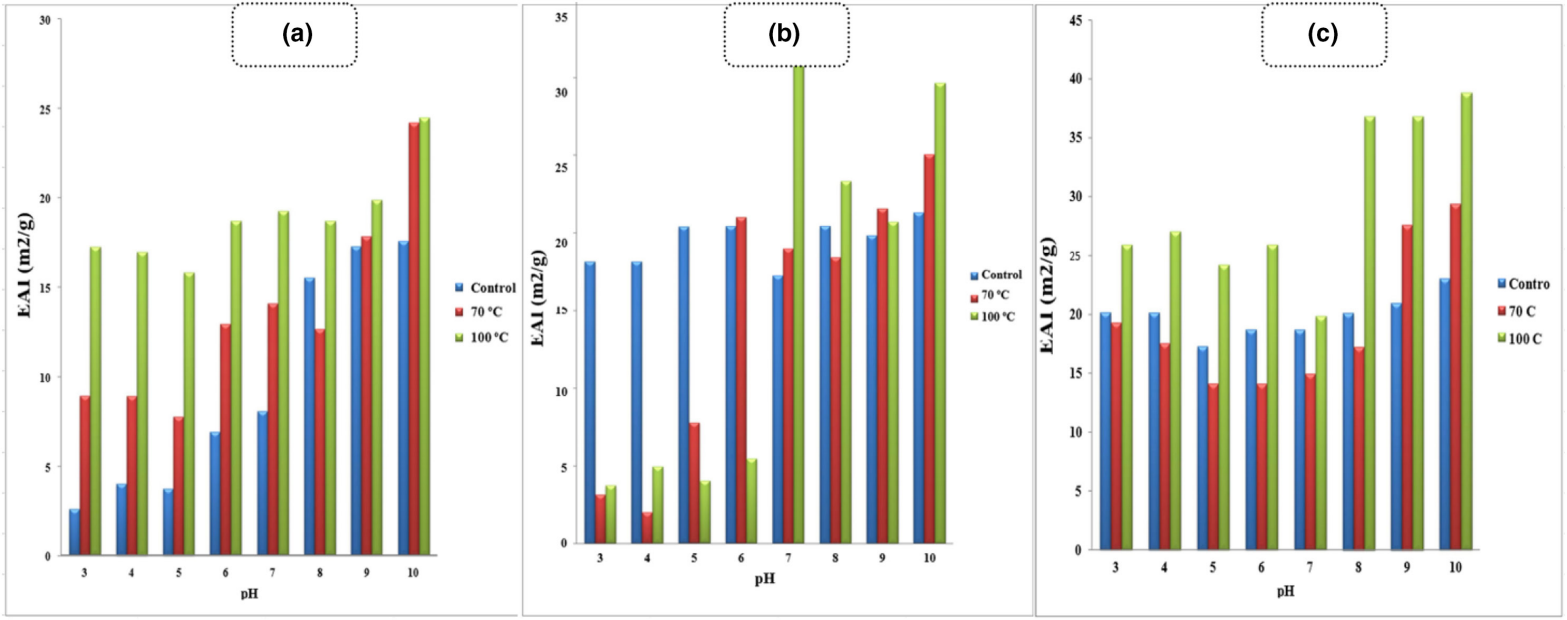
Effect of pH on the emulsion activity index (EAI) of control (■), lactosylated at 70°C for 2 h (■), and lactosylated at 100°C for 2 h (■) in 0.15 M NaCl for casein (a), whey protein (b), and total milk protein (c).

Emulsifying properties are related to the protein's ability to diffuse across the oil–water interface, unfolding and orienting, so that the hydrophobic groups approach the oil, while the hydrophilic groups approach the water phase. Figure 3 shows the effect of pH on the EAI of control caseins, which was low at pH values 6 and lower, and this is related to pH values which are close to the isoelectric point of cow milk caseins (pH = 4.6).

Lactosylation at 70 and 100°C increased the EAI at all pH values, especially at pH below 6, and the activity was higher for casein samples treated at 100°C. This could be related to the conjugation of carbohydrate to these proteins which led to increase their solubility at low pH values. Also, cross‐linking of proteins resulting from MR may change the surface properties of caseins and lead to an increase in the EAI. Jiang and Zhao (2011) reported that they measured the emulsifying activity of three protein samples and recorded 90.3, 100.9, and 70.6 m^2^/g for EAI in the original casein, modified casein product, and cross‐linked casein, respectively. A 22% decrease in the EAI of cross‐linked casein and a 12% increase in the modified product were observed compared to the original casein.

#### 3.3.2 Emulsion properties of whey proteins

Whey proteins adsorb rapidly the oil–water interface forming emulsions (Thompson et al., 2009). In Figure 3, the result of EAI of control whey proteins, whey proteins lactosylated at 70°C, and whey proteins lactosylated at 100°C for 2 h is summarized. At acidic pH values (5 and lower), the EAI of control whey proteins was higher than those of whey proteins heated at 70 and 100°C for 2 h. This is related to the high hydrophilicity of lactosylated proteins, resulting from bonding lactose, which reduces their ability to interact with the oil phase and lose their ability to diffuse into the water–oil interface (Flanagan et al., 2003).

At pH values 7 and higher, the EAI of lactosylated whey proteins increased and this fact could be related to the increase of their hydrophobicity as a result of some of the whey proteins such as lactoferrin and immunoglobulin reaching their isoelectric point (Swaisgood, 2003).

#### 3.3.3 Emulsion properties of total milk proteins

Figure 3 Shows the effect of pH on EAI of total milk proteins. The EAI of total milk proteins lactosylated at 100°C for 2 h was higher compared with the EAI of control milk proteins and total milk proteins lactosylated at 70°C at all pH values between 3 and 10. This result can be explained by the higher carbohydrate content in these samples which increases their ability to bind with water and increasing the ability to adsorb on the water–oil interface. This result is in accordance with the results of Aminlari et al. (2005). They found that the emulsifying activity of milk proteins increased after MR with dextran.

#### 3.3.4 Foaming properties

**FIGURE 4:**
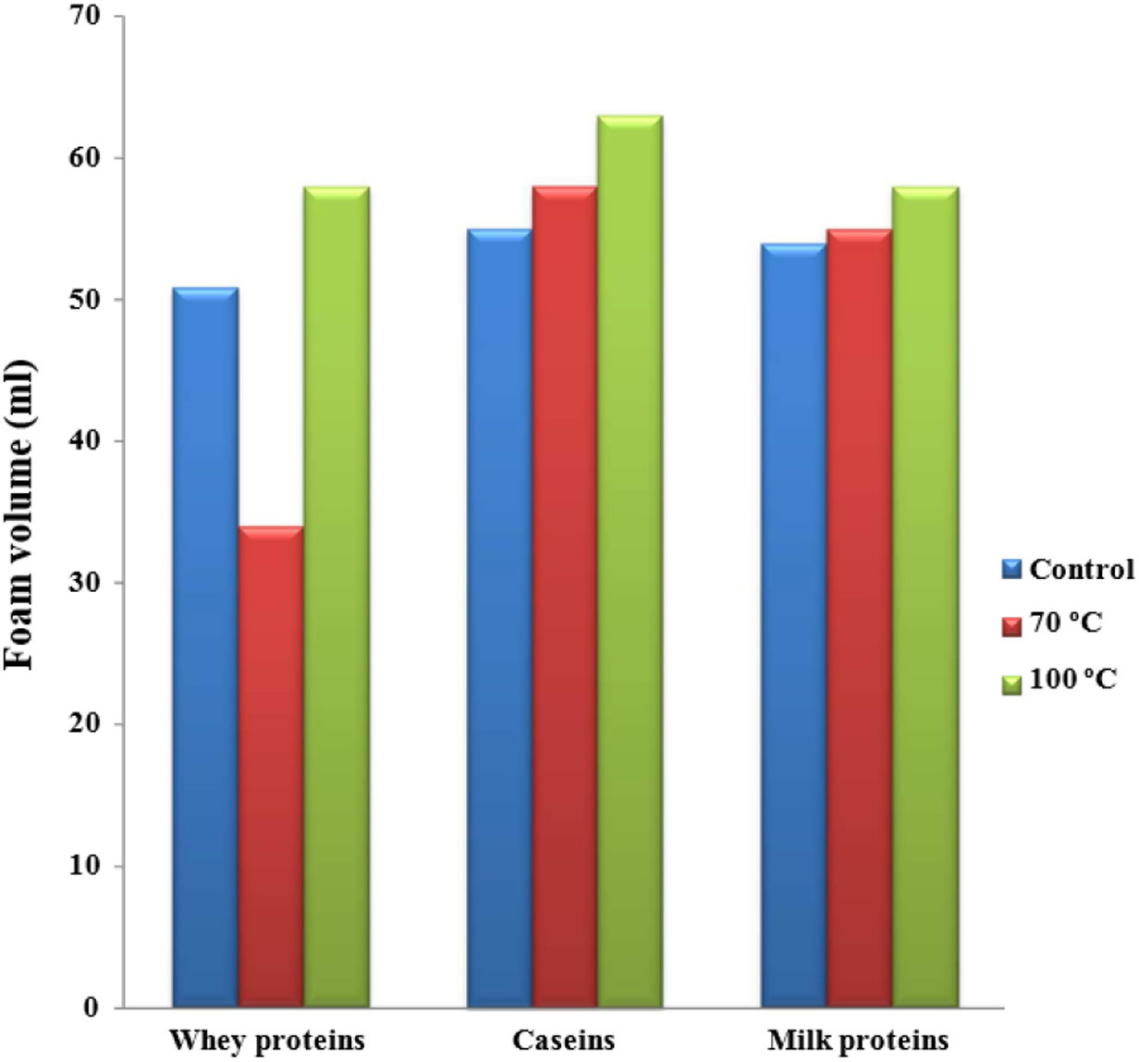
Effect of lactosylation (70 and 100°C for 2 h) on the foam volume of caseins, whey proteins, and total milk proteins.

Foam is known as a two‐phase system in which a distinct gas bubble phase is surrounded by a continuous liquid laminar phase. Due to the large liquid–gas surface area, foam requires energy to produce and is essentially unstable. Foam consists mostly of air and is recognized by low density and high viscosity, surface area, and surface energy. The effect of lactosylation on the foaming properties of caseins, whey proteins, and total milk proteins is summarized Figure 4. Foam volume of caseins, whey proteins, and total milk proteins in lactosylated at 100°C was 63, 58, and 58 ml respectively. Also, the foam volume of caseins, whey proteins, and total milk proteins lactosylated at 70°C for 2 h was 58, 34, and 55 ml, respectively, while 55, 50.9, and 54 ml were obtained for control caseins, control whey proteins, and control total milk proteins, respectively.

These differences in foaming volume may be related to heat treatment which leads to an increase in lactose conjugations to proteins and the increase in protein cross‐linking as a result of MR development (Al‐Saadi et al., 2013). This result is similar to the results of Hiller and Lorenzen (2010) which show an increase upon Maillard modification with pectin by emulsifying activity of milk proteins (up to +1242%) and dextran (up to +513%), probably due to superior water‐binding properties of these glycoconjugate proteins in oil‐in‐oil emulsions.

Jiang and Zhao (2011) evaluated the foaming properties of three casein products. The foaming activities of the original casein, modified casein product, and cross‐linked casein were 75.1%, 81.5%, and 62.3%. The foaming activity of cross‐linked casein decreased by 17% compared with original casein; these indices of the modified casein product increased by about 8.5%.

### 3.4 Effect of Maillard reactions on some physiochemical properties of cow milk powder yoghurt

The effect of lactosylation of whole milk powder at (70 and 100°C for 2 h) on the physiochemical properties such as pH, viscosity, spontaneous whey separation (SWS), hardness, and WHC of yoghurt is summarized in Table 1. The pH of control yoghurt, yoghurt treated with MTGase, yoghurt produced by whole milk powder lactosylated at 70°C, yoghurt produced by milk powder heat lactosylated at 70°C with MTGase, yoghurt produced by whole milk powder lactosylated at 100°C, and yoghurt produced by whole milk powder lactosylated at 100°C for 2 h with MTGase were 4.44, 4.43, 4.6, 4.5, 4.58, and 4.62, respectively, and these values decreased to 4.27, 4.3, 4.26, 4.19, 4.3, and 4.2, respectively, after 21 days of storage at 7 ± 1°C.

**TABLE 1:** Effect of Maillard reaction (MR) and Microbial Transglutaminase (MTGase) on some physiochemical properties (pH, viscosity, spontaneous whey separation [SWS], hardness, and water‐holding capacity [WHC]) of yoghurt during storage at 7 ± 1°C for 21 days.

Storage period (days)	Type	pH	Viscosity (cp)	SWS (ml)	Hardness (g)	WHC (%)
1st	Control	4.44 ± 0.03^a^	581.6 ± 14.5^a^	0.35 ± 0.03	71.5 ± 3.4^a^	54.4 ± 3.3^a^
	Yoghurt MTGase	4.43 ± 0.04^a^	737.3 ± 23.7^b^	0	138.5 ± 5.5^b^	55.93 ± 3.2^a^
	70°C	4.6 ± 0.03^b^	698 ± 19.8^c^	0.55 ± 0.04	61.5 ± 3.3^c^	54.3 ± 2.4^a^
	70°C & MTGase	4.5 ± 0.03^c^	1164.6 ± 33.7^d^	0	151.5 ± 4.5^d^	57.76 ± 3.5^a^
	100°C	4.58 ± 0.04^b^	709.6 ± 21.1^b^	0.2 ± 0.03	74.5 ± 3.9^a^	57.71 ± 2.2^a^
	100°C & MTGase	4.62 ± 0.05^b^	841.6 ± 34.4^e^	0	158.5 ± 4.9^d^	61.52 ± 2.7^b^
21st	Control	4.27 ± 0.03^A^	1272.7 ± 45.4^A^	0	130.0 ± 5^A^	51.62 ± 2.7^A^
	Yoghurt & MTGase	4.3 ± 0.02^A^	1116.7 ± 43.3^A^	0	167.0 ± 5.5^B^	55.34 ± 2.4^A^
	70°C	4.26 ± 0.03^A^	1721.3 ± 47.7^B^	0	136.0 ± 4.2^A^	55.72 ± 2^A^
	70°C & MTGase	4.19 ± 0.02^B^	1600.3 ± 54.3^C^	0	174.0 ± 6.2^B^	63.47 ± 3^B^
	100°C	4.3 ± 0.03^A^	1766.7 ± 56^B^	0	129.0 ± 4.6^A^	63.49 ± 2.3^B^
	100°C & MTGase	4.2 ± 0.02^B^	1860.3 ± 55.1^D^	0	207.0 ± 8.7^C^	65.93 ± 3.2^B^

*Note*: Different letters indicate significant differences (*α* = 0.05).

The pH of the MTGase‐treated samples lactosylated at 100°C for 2 h was slightly higher compared to the other samples after 1 day. In accordance with these results, Lorenzan et al. (2002) indicated that the time of fermentation prolonged for all samples treated with MTGase compared to the control sample. These values are similar to the results of Ozer et al. (2007), since they observed that the pH of yoghurt samples produced by cow milk powder were between 4.4 and 4.1. This decrease in pH values during storage time is related to yoghurt starter activity that converts lactose to lactic acid and formation of formic acid during the MR.

Viscosity is an important factor that can be reliably used to compare the quality of yoghurt samples prepared under different conditions. Viscosity improvement depends on enzyme concentration and incubation time. In addition to the incubation conditions, the samples treated with MTGase have more favorable rheological properties compared to the control samples. The higher viscosity for samples manufactured from lactosylated whole milk powder is related to the effect of heat treatment which induced the aggregation of milk proteins which leads to an increase in the viscosity of yoghurt samples.

For the identification of protein cross‐linking, SDS‐PAGE was used. Figure S2 shows the enzymatic cross‐linking of protein in set yoghurt produced after first and 21st. As shown in Figure S2, the rate of cross‐linking is clear in treated compared with control samples. The band intensity increased upon increasing the enzyme concentration in control MTGase, 70°C MTGase, and 100°C MTGase, respectively. An increase in molecular mass of (*α*s and *β*) casein fractions observed was due to the effect of cross‐linking reaction elicited by MTGase addition as described by Wroblewoska et al. (2009) previously, and the amount of protein monomers in yoghurt increased upon enzymatic modification in MTGase‐treated yoghurt sample compared to the control sample. In Figure S2, it can be found that the cross‐linking of milk proteins increased by enhancing the whole milk powder's lactosylation temperature and treatment with MTGase.

This increase in the cross‐linking of milk proteins, which occurs through MR, affects the physiochemical and sensory properties of the dairy product (Al‐saadi & Deeth, 2011). SWS was determined by measuring the volume of whey separation on the top of yoghurt sample. SWS was very low in all yoghurt samples on the first day of storage and became 0 after 21 days of storage at 7 ± 1°C. These results are similar to the founded results by Salvador and Fiszman (2004).

Hardness is the necessary force to attend to deformation in the gel, its common measure to indicate the strength of gel network. In Table 1, it can be observed that the hardness is increased after 21 days of storage at 7 ± 1°C for all samples. MTGase‐treated yoghurt samples may be attributed to increase of cross‐linking caused by the simultaneous addition of MTGase with the starter. Therefore, enzyme addition by this method leads to a greater chance for cross‐linking which occurs during the coagulation process and continued during storage. Yuksel and Erdem (2010) found similar results for yoghurt with active MTGase and they illustrated that this type of yoghurt has a higher initial hardness which continued to increase during storage time. The highest value of hardness is recorded in yoghurt samples prepared by whole milk powder lactosylated at 100°C for 2 h and treated with MTGase after 21 days of storage at 7 ± 1°C compared to the other samples. The higher hardness of yoghurt samples produced by whole milk powder lactosylated at 70 and 100°C for 2 h and treated with MTGase compared to the yoghurt samples produced by milk treated with MTGase (without heat treatment) is related to the role of MR in cross‐linking milk proteins and strengthening the protein network of yoghurt, and as a result, a higher ability to retain water (Hiller & Lorenzen, 2011).

Water‐holding capacity means the capacity of a gel to hold water, and with the increase of WHC the quantity of water in the gel will be increased. WHC of the yoghurt was determined by estimating the intensity of the syneresis phenomena after centrifugation. The best WHC was recorded for the yoghurt samples with 100°C MTGase. Table 1 shows that WHC after 1 day of produced yoghurt were 54.4%, 55.93%, 54.3%, 57.76%, 57.71%, and 61.52% for control yoghurt, yoghurt treated with MTGase, yoghurt produced by whole milk powder lactosylated at 70°C, yoghurt produced by milk powder lactosylated at 70°C with MTGase, yoghurt produced by whole milk powder lactosylated at 100°C, and yoghurt produced by whole milk powder lactosylated at 100°C for 2 h, respectively, and these values changed to 51.62%, 55.34%, 55.72%, 63.47%, 63.49%, and 65.93%, respectively, after 21 days of storage at 7 ± 1°C. In general, the combination of MTGase and heat treatment in (100°C) significantly increased the WHC in yoghurt produced by whole milk powder compared to control samples.

The decrease in WHC of control yoghurt during storage is due to enhancing its acidity which increases the positive charge presumably reducing intermolecular interactions which result in the formation of an open (porous) structure leading to a decrease in WHC of yoghurt (Meydani et al., 2019). The increase of WHC of yoghurt samples as a result of lactosylation is related to the effect of MR on inducing cross‐linking of milk proteins, which makes the protein network stronger and their ability to bind water higher (Hiller & Lorenzen, 2011). Also, increase in WHC of yoghurt samples treated with MTGase can be due to this enzyme activity in the cross‐linking of milk proteins (Yuksel & Erdem, 2010).

Hannß et al. (2018) studied the stability of acidic gels prepared from original and glycosylated casein after short‐term storage with gel strength and WHC measured through texture analysis and the centrifugal method. They claimed that the gel strength of gels prepared from heated caseins did not increase significantly compared to the control. The gel strength values of glycosylated casein samples spontaneously decrease below the initial gel strength values during prolonged heating times, which indicate instability of the gel network. Also, a prominent increase in WHC from a minimum of 61% to a maximum of 87% was shown for both casein–lactose mixtures after glycosylation. Although WHC decreased to a certain extent for a short period of dry‐heating in the presence of lactose, in the absence of lactose, the WHC of acid casein gels was not affected by casein heating. Meydani et al. (2019) studied the influence of the MR on the properties of cold‐set whey protein and maltodextrin binary gels. The Maillard conjugation of whey proteins and maltodextrin increased the WHC of binary gels at both total solid content series, so that the WHC of whey protein isolate (WPI)–maltodextrin conjugate gel at 200 mg/ml total solid content was comparable to that of the control WPI gel (*p* > .05). The increased WHC of binary gels due to the MR is correlated with the less porous microstructure of the corresponding gels.

### 3.5 Effect of Maillard reaction on the sensory properties of cow milk powder yoghurt

**FIGURE 5:**
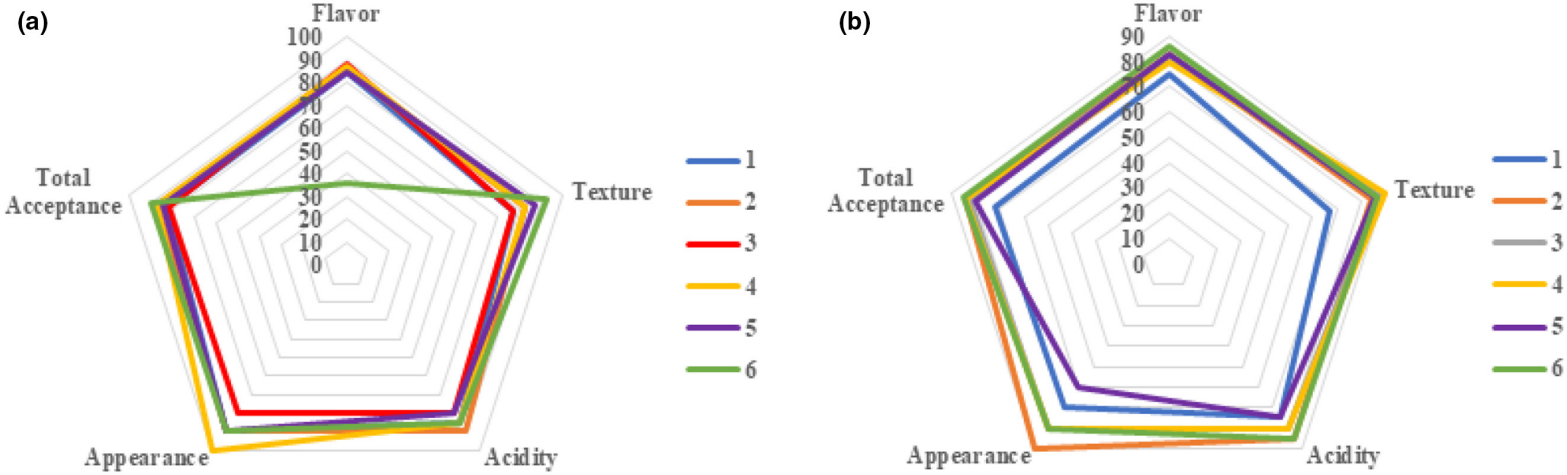
Spider plot for describing the effect of Maillard reaction (MR) and Microbial Transglutaminase (MTGase) on the sensory properties of yoghurt during storage at 7 ± 1°C for first (a) and 21st (b) days. Control yoghurt (1), yoghurt treated with MTGase (2), yoghurt produced by whole milk powder lactosylated at 70°C for 2 h (3), yoghurt produced by whole milk powder lactosylated at 70°C with MTGase for 2 h (4), yoghurt produced by whole milk powder lactosylated at 100°C for 2 h (5), and yoghurt produced by whole milk powder lactosylated at 100°C with MTGase for 2 h (6).

Sensory evaluation was carried out for yoghurt samples to estimate the acceptability of yoghurt samples by 12 panelists. The panelists were invited to evaluate the flavor, texture, acidity, appearance, and total acceptance of yoghurt after 1 and 21 days of storage. Figure 5 shows that the total score of control yoghurt was 83, while the total scores for yoghurt treated with MTGase, yoghurt manufactured from whole milk powder lactosylated at 70°C, yoghurt manufactured from whole milk powder lactosylated at 70°C with MTGase, yoghurt manufactured from whole milk powder lactosylated at 100°C, and yoghurt manufactured from whole milk powder lactosylated at 100°C with MTGase were 87, 82, 87, 85, and 90, respectively, at the first day of storage at 7 ± 1°C and these values changed to 72, 84, 83, 83, 80, and 85 after 21 days of storage at 7 ± 1°C, respectively. This decrease in sensory evaluation score after 21 days of storage was mainly maybe due to the increase in their acidity (Ozer et al., 2007).

The effect of aggregation of milk proteins using TGAse and MR on Ca^2+^ milk gel has been studied by Alfayadh et al. (2018) who found that the total scores were 80.6, 75.3, 84.3, and 83.1 for the control gel, Maillard‐modified milk, milk treated with TGAse, and milk treated with Maillard/TGAse after 1 day of storage at 7°C, which changed to 80, 78.5, 90.5, and 89.5, respectively, at the end of the storage period at 7°C. Among all milk calcium gels, the gel prepared from Millard's modified milk obtained the lowest sensory score from the panelists, which is probably due to the separation of whey in the top layer of the gel.

## 4 CONCLUSIONS

Heat treatment of milk proteins in the presence of lactose increased the lactose ratio in these proteins and increased the cross‐linking between these proteins, at 100°C the heat treatment of all milk protein samples was more effected than at 70°C for 2 h. Lactosylation of caseins, whey proteins, and total milk proteins through Millard reaction (MR) increased their solubility at low pH values. Lactosylation of caseins and total milk proteins by a prolonged heat treatment increased their EAI, the viscosity, hardness, WHC, and sensory properties of yoghurt during storage for 21 days. Casein, whey protein, and total protein were modified by MTGase in the presence of glucosamine to prepare a cross‐linked and glucosamine‐conjugated casein product. The results showed that the use of MTGase improved some of the functional properties of the modified casein product, for example, it increased the solubility, improved the emulsifying property, significantly improved the textural properties, and reduced the surface hydrophobicity which may be due to increased water‐binding properties of casein glycoconjugates. The enzymatic glycosylation method of MTGasee may be used as a practical tool to improve some functional characteristics of food proteins.

## 5 ACKNOWLEDGEMENTS

Salahaddin University Erbil‐Iraq is appreciated for supporting this research in terms of supplying laboratory materials and equipment.

## Supporting information


Figure S1
