# Supplementary material for: Functional and physiochemical properties of the yoghurt modified by heat lactosylation and microbial transglutaminase cross‐linking of milk proteins
Source: Food Sci Nutr. 2022 Oct 21;11(2):722–32. doi: 10.1002/fsn3.3108 (PMC9922141; doi:10.1002/fsn3.3108)
Supplement: Supplementary file 1 — Figure S1 [file FSN3-11-722-s001.docx]

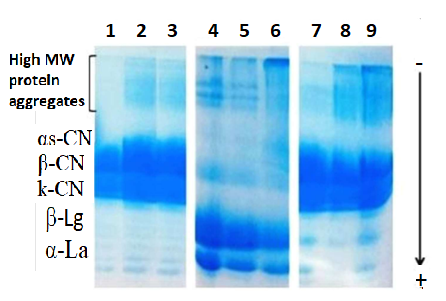


##### Fig S1. SDS-PAGE of the heat treated milk proteins 1-control caseins, 2-caseins lactosylated at 70 ºC for 2 h, 3-caseins lactosylated at 100 ºC for 2 h, 4-control whey proteins, 5-whey proteins lactosylated at 70 ºC for 2 h, 6-whey proteins lactosylated at 100 ºC for 2 h, 7-control total milk proteins, 8- total milk proteins lactosylated at 70 ºC for 2 h and 9- total milk proteins lactosylated at 100 ºC for 2 h.


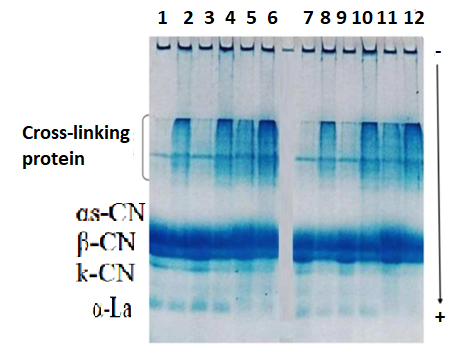


##### Fig S2. SDS-PAGE of yoghurt samples (1 and 7) control yoghurt (2 and 8) yoghurt + MTGase (3 and 9) yoghurt produced by whole milk powder lactosylated at 70 ºC for 2 h (4 and 10) yoghurt produced by whole milk powder lactosylated at 70 ºC for 2 h + MTGase (5 and 11) yoghurt produced by whole milk powder lactosylated at 100 ºC for 2 h (6 and 12) yoghurt produced by whole milk powder lactosylated at 100 ºC for 2 h + MTGase (samples 1, 2, 3, 4, 5 and 6 are for first day of storage and samples 7, 8, 9, 10, 11 and 12 are for 21^th^ day of storage at 7 ºC.
